# Predictors of prospective suicide attempts in a group at risk of personality disorder following self-poisoning

**DOI:** 10.3389/fpsyt.2023.1084730

**Published:** 2023-06-09

**Authors:** Lionel Cailhol, Mariève Marcoux, Anjali Mathur, Antoine Yrondi, Philippe Birmes

**Affiliations:** ^1^Department of Psychiatry, Institut Universitaire de Santé Mentale de Montréal, CIUSSS of East Montreal, University of Montreal, Montreal, QC, Canada; ^2^Department of Psychology, University of Montreal, Montreal, QC, Canada; ^3^Department of Brief therapy, University Hospital Center of Toulouse (CHU Toulouse), Toulouse, Occitanie, France; ^4^Department of Psychiatry and Medical Psychology, University Hospital Center of Toulouse (CHU Toulouse), Expert Centre for Treatment-Resistant Depression FondaMental, ToNIC Toulouse NeuroImaging Centre, Toulouse University, Toulouse, Occitanie, France; ^5^Department of Psychiatry, Psychotherapies and Art Therapy, University Hospital Center of Toulouse (CHU Toulouse), ToNIC Toulouse NeuroImaging Centre, Toulouse University, Toulouse, Occitanie, France

**Keywords:** personality disorder, alexythimia, suicidal behavior (SB), suicide, suicidal ideation (SI), self-poisoning nonlethal suicide attempt, emergency department

## Abstract

**Background:**

Patients with personality disorder (PD) are at risk for suicidal behavior and are frequently admitted for this reason to emergency departments. In this context, researchers have tried to identify predictors of their suicidal acts, however, the studies have been mostly retrospective, and uncertainty remains. To prospectively explore factors associated with suicide attempts (SA) in individuals screened for PD from the ecological context of emergencies.

**Methods:**

Patients were recruited from two emergency departments after a self-poisoning episode (*n* = 310). PDQ-4+ (risk of PD), TAS-20 (alexithymia), SIS (suicidal intent), H (hopelessness), BDI-13 (depression), AUDIT (alcohol consumption), and MINI (comorbidity) questionnaires were completed. SA over the subsequent two years were identified by mailed questionnaires and hospitals’ active files. Logistic regression analyses were performed.

**Results:**

Having a previous suicidal attempt was linked to a 2.7 times higher chance of recurrence after 6 months, whereas the TAS-20 showed a 1.1 times higher risk at 18 months (OR = 1.1) and the BDI at 24 months (OR = 1.2). Each one-unit increment in TAS-20 and BDI-13 scores increased the risk of SA by 9.8 and 20.4% at 18 and 24 months, respectively.

**Conclusion:**

Some clinical features, such as alcohol dependence, suicide intent, and hopelessness, may not be reliable predictors of SA among PD patients. However, in the short term, previous SA and, in the long term, depression and alexithymia may be the most robust clinical predictors to consider in our sample of patients with self-poisoning SA.

**Clinical trial registration**: [ClinicalTrials.gov], NCT00641498 24/03/2008 [#2006-A00450-51].

## Introduction

In the general population, at least 8% of individuals (1) may have a personality disorder (PD). PDs cause distress and alter functioning, which often leads to suicidal behavior (SB). Up to 57% of people who die by suicide are diagnosed with PD (2). In emergency departments, the prevalence of patients admitted for suicide attempts (SA) or completed suicide has been reported to be 35% (3, 4). Among the 10 recognized PDs, borderline PD is the one that is known to spark the most SA (5). Indeed, after a 10-year follow-up, 13% of borderline patients still reported having SA, compared to 3% for the other forms of PD combined (6).

Factors related to SA or suicide have been assessed, often regardless of diagnosis. Assessing suicidal risks is different from assessing suicidal emergency. Emergency refers to the likelihood that a person will commit suicide within few days. Risk refers to the likelihood that an individual will act throughout their lifetime based on individual factors and life circumstances. Certain risk factors have been linked to both SA and suicide, such as previous SA, depression, psychiatric treatment, low income, and low education (7). Other factors distinguished the two concepts: SA have been linked to a younger age and to social isolation and more frequently performed by women and by drug intoxication (7–9). Suicide is more frequently performed by men, is more frequently performed by hanging or firearm, and has been linked to an older age, alcohol abuse and type B personality disorder (7–10). Nonetheless, Qin’s study (11) demonstrated that risk factors vary considerably depending on the individual’s diagnosis. In this respect, it is logical to verify whether general known predictors are well maintained in individuals with PD. Actually, it is often a challenge for clinicians to differentiate among patients who have PD those who have chronic suicidal thoughts and those of high risk of suicide (12). One of the crucial factors in this distinction is the presence of suicidal ideation (SI), but obtaining access to this information can be difficult, and there are other significant risk factors to consider as well.

In this line of research, various studies have considered a PD diagnosis to establish suicide risk factors. However, these studies have used mostly retrospective methods, and questions remain. First, emotional dysregulation has been related many times to PDs (13), and ineffective management leads to SA (14). This correlation could underlie alexithymia, the inability to identify, express and describe one’s feelings. Since those with alexithymia are unable to encounter their true emotions, they remain negatively affected and may use inappropriate regulation methods, such as SA or suicide, to express them. Second, suicidal intent is defined as both the desire to die and the belief that death will result from a self-inflicted wound. Generally, the higher the suicidal intent is, the better the predictor of subsequent SB (15, 16). Compared to other psychiatric diagnostics, patients with PD had significantly lower suicidal intent and higher hopelessness mean scores after self-poisoning acts (17).

Thus, we have on the one hand an extensive literature on suicidal risk factors and on the other hand several longitudinal studies of patients with PD. However, there are no prospective studies at the level of the emergency room psychiatrist who needs to know what the suicide risk factors are among patients admitted for SA and with probable PD. Our goal was to prospectively assess predictors of SA in subjects at risk of PD.

## Methods

### Trial design

This project derives from a multicenter, prospective, and randomized clinical trial (“French Crisis”). Preliminary results involving alcohol and repeated self-harm have been published (18).

### Participants

The inclusion criteria were (1) having a self-poisoning episode (intentional act of ingesting a toxic substance or a harmful chemical with the intent of harming oneself), (2) receiving emergency medical care, (3) experiencing normal consciousness (Glasgow score = 15), and (4) being ≥18 years of age. The exclusion criteria were (1) insufficient comprehension of French, (2) residence far from the recruitment center to ensure proper tracking in the databases of the participating hospitals, (3) inability to give consent or under a protective measure (e.g., guardianship or curatorship), and (4) visual impairment making reading impossible. Formally, 606 participants were included.

Recruitment took place from March 2007 to June 2009, Mondays to Fridays, in two French emergency units (Toulouse University Hospital and Brive Hospital). Once the resuscitation care and the consultation by the psychiatrist on duty were completed, the patients were directly evaluated in their room. The interviews lasted between 1 and 1.5 h. Various questionnaires were administered. For the next 2 years, the patients were mailed a questionnaire every 6 months with a prepaid envelope. The randomization was prepared by closed envelopes. It was balanced on the center and on possible hospitalization. In each center, two sets of envelopes were available: one set for patients requiring hospitalization and one set for those returning home. The initial interview, conducted by master level psychological trainees, occurred prior to randomization, and the follow-up was conducted via self-administered questionnaire, ensuring the blinding process. In the present paper, this distinction was not considered, and all subjects were included in the analyses, regardless of randomization. As a result, we do not have a separate control group for comparison.

### Interventions

During the study, the patients in the control intervention benefited from the usual follow-up (hospitalization, follow-up in the community and medication). The patients in the experimental group benefited from the usual treatment and a psychological interview from the emergency room with outpatient follow-up in a center specializing in suicidology. We lack specific details regarding the treatment as usual group, which was observed naturally. The type of treatment received by patients varied and could have been either outpatient or inpatient. Patients may have received treatment from a psychologist, general practitioner, or psychiatrist. The use of psychotropic medications was common, and patients may have also undergone various forms of psychotherapy.

### Instruments

Participants could report whether they had made a SA since recruitment through 6 (main outcome), 12, 18, and 24 months. An objective verification was also made using the hospital’s active file where the patient had been initially enrolled.

Information was obtained regarding age, sex, marital status, education level, employment status, suicidal history, consultation with a health professional in the past 6 months, and finally, use of medication in the past 6 months (only antidepressants were targeted in our secondary analysis).

The Diagnostic Personality Questionnaire (PDQ-4+) (19–21) is a self-report screening tool for PD. It contains 99 true/false items. The total score is calculated by adding up all “true” responses, each worth 1 point, while excluding validity questions (#12, 25, 38, 51, 64, and 76). The threshold defined to assess the possible presence of one or more PDs was ≥28 points (22). Cronbach’s *α* for our sample was 0.91.

The Toronto Alexithymia Scale (TAS-20) (23, 24) is a self-administered Likert-type scale assessing difficulty in identifying and distinguishing emotional states, difficulty in verbalizing them, and reduced fantasy life. It consists of 20 items rated from 1 (complete disagreement) to 5 (complete agreement). The points are summed after reverse scoring items #4, 5, 10, 18, and 19. The total score can range from 20 to 100, and a score of ≥61 indicates alexithymia. Cronbach’s *α* for our sample was 0.75.

The Suicidal Intent Scale (SIS) (25, 26) contains an objective section and a subjective section that assess the intensity of the desire to die. There are 15 questions, each graded from zero to two. The total score is obtained by adding each item and can range from 0 to 30. The severity threshold according to Conner et al. (27) is labeled low from 0 to 9, medium from 10 to 15, and high from 16 and above. Cronbach’s *α* for our sample was 0.77.

The Hopelessness Scale (BHS) (28) is a self-administered measure of pessimism. It consists of 20 true/false items. One point is attributed to items #2, 4, 7, 9, 11, 12, 14, 16, 17, 18, and 20 if rated true, and items #1, 3, 5, 6, 8, 10, 13, 15, and 19 receive one point if rated false. The total score can range from 0 to 20. A score of 9 or higher indicates increased hopelessness. Cronbach’s *α* for our sample was 0.90.

The short version of the Beck Depression Inventory (BDI-13) (29) is a self-administered scale that evaluates subjective aspects of depression. There are 13 questions rated on a scale from 0 to 3. The total score is obtained by adding each item and can vary between 0 and 39. The following thresholds were used: 0–4, no depression, 4–7, mild depression, 8–15, moderate depression, and 16 or more, severe depression. Cronbach’s *α* for our sample was 0.83.

The Alcohol Use Disorders Identification Test (AUDIT) (29) consists of ten questions: questions 1–3 assess alcohol consumption, questions 4–6 assess drinking-related behaviors, questions 7–8 assess harm, and questions 9–10 assess drinking-related problems. Each question is worth 0 to 4 points. The total score is obtained by adding the points for each item and can range from 0 to 40. A total score ≥8 indicates hazardous use. Cronbach’s *α* for our sample was 0.92.

The Mini International Neuropsychiatric Interview (M.I.N.I. 5.0.0) (30) is a structured interview for diagnostic purposes. Seventeen psychiatric disorders can be explored based on 120 questions organized in a logical tree. Only major depression and alcohol abuse were targeted in our secondary analyses. The kappa reliability coefficients were good and ranged from 0.88 to 1.00.

At the first visit, only the MINI and PDQ-4+ were utilized, while self-administered questionnaires such as the TAS, BDI, BHS, AUDIT and SIS were employed at every measurement interval. Whenever the research team obtained a new questionnaire, they meticulously evaluated it for signs of suicidal ideation (SI). If deemed necessary, a psychiatrist from the team would immediately reach out to the subject and make contact.

### Sample size

This report focuses on participants who completed the PDQ4+ questionnaire, which distinguishes individuals at risk of PD from those who are not. We estimated that 50% of subjects recruited could have at least one PD and that the drop-out rate could be close to 50%. With a power of 80% and alpha risk at 5%, the target sample size was calculated to be 152 to detect risk ratio at 1.5 (Fleiss with correction for continuity).

### Statistical methods

IBM SPSS Statistics 26 software was used (IBM Corp, New York, United States), with an alpha significance level set at 0.05.

1. **Missing data**. Little’s test was performed to check whether the missing data followed a completely random pattern. Since it was, missing responses on the five questionnaires (PDQ4-+, TAS-20, SIS, H, BDI-13, AUDIT) were replaced using five multiple imputations. By variable, the amount of missing data ranged from 0.3 to 11.4%. If participants did not complete a questionnaire, it was not imputed. A pairwise deletion technique was used when possible.
2. **Group differences** (SA – no attempt). *t*-tests (or Mann–Whitney tests if normality and/or homogeneity of variance was not respected) and Chi-2 (or Fisher and likelihood ratio if minimum frequencies per class were not respected) were performed to identify differences between collected variables. Statistically significant variables were identified as potential predictors.
3. **Logistic regression analyses**. Potential predictors were included in a stepwise conditional ascending logistic regression due to the exploratory nature of the study. Steps 2 and 3 were performed for each follow-up period.

## Results

**Figure 1:**
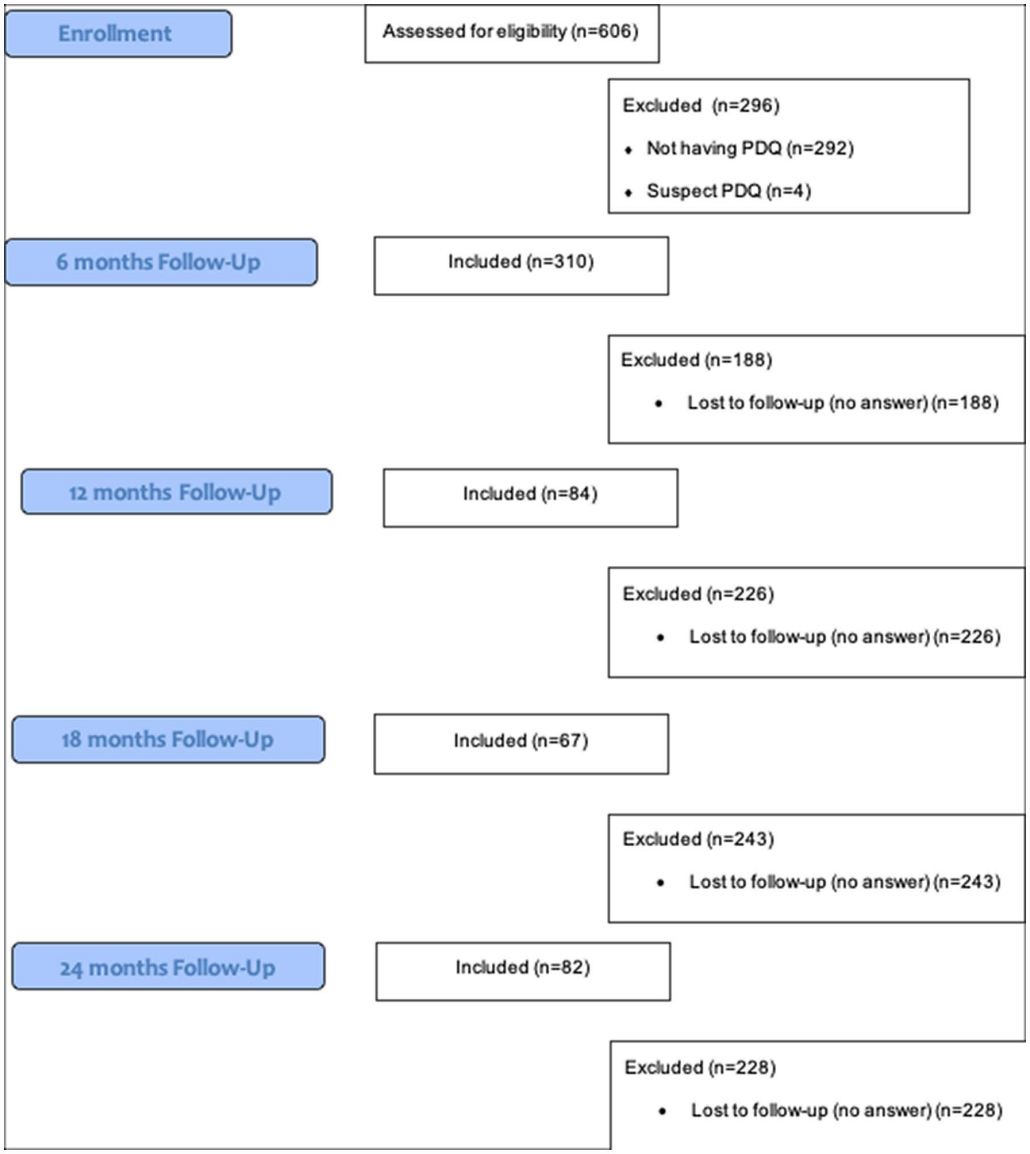
Recruitment and follow-up flow-chart.

In total, 310 participants out of the 606 completed the PDQ4-+ (Flow-chart, Figure 1). Four participants had to be excluded from the analyses because of their answer to question 76 (“I have lied a lot on this questionnaire”). Using valid questionnaires, 243 were identified as being at risk of PD. Women comprised 76% of the sample (*n* = 184; Table 1). On average, the participants were at risk of five to six types of PD. Overall, there were 192 individuals identified as being at risk for avoidant PD (14%); 190, borderline PD (14%); 182, depressive PD (13%); 168, paranoid PD (12%); 155, obsessive–compulsive PD (11%); 110, schizotypal PD (8%); 94, negativistic PD (7%); 81, schizoid PD (6%); 65, dependent PD (5%); 64, narcissistic PD (5%); 59, histrionic PD (4%); and 16, antisocial PD (1%). According to MINI, 45% (*N* = 55) of the initial participants reported suffering from depression, and 5.3% (*N* = 6) reported substance abuse. Additionally, 67.2% (*N* = 82) reported having previously been prescribed an antidepressant, and 44.3% (*N* = 51) reported a previous SA.

**Table 1:** Comparison of sociodemographic characteristics between the two groups (no SA, SA) at each follow-up time.

Follow-up time	Variables	No SA	SA	Test value (df)	*p*
6 months	**Sex**: number (%)			–^c^	0.795
	Male	20 (21.3)	5 (17.9)		
	Female	74 (78.7)	23 (82.1)		
	**Age**: mean (SD)	39.7 (13.7)	39.0 (13.9)	1272.000 (120)^b^	0.789
	**Marital** status:			5.207 (4)^b^	0.267
	Single	17 (19.5)	6 (22.2)		
	Married	28 (32.2)	7 (25.9)		
	Divorced/separated	30 (34.5)	6 (22.2)		
	Widowed	4 (4.6)	1 (3.7)		
	Civil partnership/cohabitation	8 (9.2)	7 (25.9)		
	**Children dependency**:			–^b^	0.474
	No children	58 (69.9)	16 (61.5)		
	One or more children	25 (30.1)	10 (38.5)		
	P**rofession**:			7.947 (5)^d^	0.159
	Unemployed	18 (20.7)	4 (14.8)		
	Retired	9 (10.3)	1 (3.7)		
	At home	2 (2.3)	2 (7.4)		
	Student	11 (12.6)	7 (25.9)		
	Employed	42 (48.3)	9 (33.3)		
	Military services	5 (5.7)	4 (14.8)		
	**Education**			1.462 (5)^d^	0.917
	None	5 (5.8)	1 (3.7)		
	“Brevet”	9 (10.3)	4 (14.8)		
	“CAP/BEP”	20 (23)	5 (18.5)		
	Baccalaureate	22 (25.3)	7 (25.9)		
	First cycle	14 (16.1)	3 (11.1)		
	2^nd^ and 3^rd^ cycle	17 (19.5)	7 (25.9)		
12 months	**Sex**: number (%)			–^b^	1.000
	Male	14 (20)	3 (21.4)		
	Female	56 (80)	11 (78.6)		
	**Age**: mean (SD)	38.6 (13.8)	38.7 (12.7)	−0.039 (82)^a^	0.969
	**Marital** status:			3.166 (4)^d^	0.530
	Single	21 (33.9)	3 (23.1)		
	Married	18 (29)	4 (30.8)		
	Divorced/separated	13 (21)	2 (15.4)		
	Widowed	2 (3.2)	0 (0)		
	Civil partnership/cohabitation	8 (12.9)	4 (30.8)		
	**Children dependency**:			–^b^	0.756
	No children	36 (62.1)	9 (69.2)		
	One or more children	22 (37.9)	4 (30.8)		
	P**rofession**:			1.644 (5)^d^	0.896
	Unemployed	13 (21)	3 (23.1)		
	Retired	3 (4.8)	1 (7.7)		
	At home	2 (3.2)	0 (0)		
	Student	10 (16.1)	2 (15.4)		
	Employed	29 (46.8)	5 (38.5)		
	Military services	5 (8.1)	2 (15.4)		
	**Education**			3.226 (5)^d^	0.665
	None	3 (4.9)	1 (7.7)		
	“Brevet”	6 (9.8)	2 (15.4)		
	“CAP/BEP”	12 (19.7)	1 (7.7)		
	Baccalaureate	17 (27.9)	3 (23.1)		
	First cycle	9 (14.7)	4 (30.8)		
	2^nd^ and 3^rd^ cycle	14 (23)	2 (15.4)		
18 months	**Sex**: number (%)			–^b^	1.000
	Male	11 (19.3)	2 (20)		
	Female	46 (80.7)	8 (80)		
	**Age**: mean (SD)	37.9 (14.1)	40.6 (12.4)	−0.565 (65)^a^	0.574
	**Marital** status:			7.841 (4)^d^	0.098
	Single	17 (32.7)	0 (0)		
	Married	18 (34.6)	5 (55.6)		
	Divorced/separated	12 (23.1)	2 (22.2)		
	Widowed	1 (1.9)	0 (0)		
	Civil partnership/cohabitation	4 (7.7)	2 (22.2)		
	**Children dependency**:			–^b^	0.460
	No children	31 (63.3)	4 (44.4)		
	One or more children	18 (36.7)	5 (55.6)		
	P**rofession**:			2.180 (5)^d^	0.824
	Unemployed	11 (21.6)	1 (11.1)		
	Retired	4 (7.8)	1 (11.1)		
	At home	2 (3.9)	0 (0)		
	Student	10 (19.6)	1 (11.1)		
	Employed	21 (41.2)	5 (55.6)		
	Military services	3 (5.9)	1 (11.1)		
	**Education**			6.887 (5)^d^	0.229
	None	0 (0)	1 (11.1)		
	“Brevet”	5 (9.6)	0 (0)		
	“CAP/BEP”	11 (21.2)	3 (33.3)		
	Baccalaureate	14 (26.9)	3 (33.3)		
	First cycle	11 (21.2)	1 (11.1)		
	2^nd^ and 3^rd^ cycle	11 (21,2)	1 (11,1)		
24 months	**Sex**: number (%)			–^b^	0.346
	Male	14 (19.2)	0 (0)		
	Female	59 (80.8)	9 (100)		
	**Age**: mean (SD)	42.1 (13.6)	37.7 (16.1)	278.000 (80)^d^	0.453
	**Marital** status:			1.305 (4)^d^	0.861
	Single	16 (23.9)	3 (33.3)		
	Married	23 (34.3)	3 (33.3)		
	Divorced/separated	15 (22.4)	2 (22.2)		
	Widowed	4 (6)	0 (0)		
	Civil partnership/cohabitation	9 (13.4)	1 (11.1)		
	**Children dependency**:			–^b^	0.193
	No children	50 (76.9)	4 (50)		
	One or more children	15 (23.1)	4 (50)		
	P**rofession**:			6.901 (5)^d^	0.206
	Unemployed	12 (17.9)	3 (33.3)		
	Retired	8 (11.9)	1 (11.1)		
	At home	4 (6)	0 (0)		
	Student	7 (10.4)	3 (33.3)		
	Employed	30 (44.8)	1 (11.1)		
	Military services	6 (9)	1 (11.1)		
	**Education**			4.740 (5)^d^	0.448
	None	4 (6)	1 (11.1)		
	“Brevet”	11 (16.4)	0 (0)		
	“CAP/BEP”	11 (16.4)	1 (11.1)		
	Baccalaureate	15 (22.4)	4 (44.4)		
	First cycle	12 (17.9)	1 (11.1)		
	2^nd^ and 3^rd^ cycle	14 (20.9)	2 (22.2)		

a *t*-test.

b Mann–Whitney.

c Fisher’s exact test (returns *p*-value only).

d Likelihood ratio test.

### Potential predictors of SA at 6 months

At the 6 month follow-up, 28 out of 122 subjects (22.9%) have committed a new act of self-harm. Among the potential predictors, psychiatric history, substance abuse, and antidepressant use 6 months prior to recruitment did not reveal a significant relationship with SA occurring within the first 6 months. Only the history of a SA was retained, *χ*^2^ (1, *n* = 116) = 5.19, *p* = 0.023, *φ* = 0.21, which increased the risk of reattempting suicide (OR = 2.68, 95% CI = 1.01–7.06, *p* = 0.047) (Table 2). It explained 6% of the variance in SA and correctly classified 80% of cases.

**Table 2:** Results of logistic regression analyses: variables associated with the occurrence of a SA for people at risk of PD.

Follow-up time	Risk factors	*β*	ES	Wald	*p*	Exp(*β*)	OR (95% CI)
6 months	Previous SA	0.984	0.495	3.955	0.047	2.676	1.014–7.062
	Constant	−1.872	0.380	24.292	0.000	0.154	
18 months	Total TAS-20	0.094	0.045	4.329	0.037	1.098	1.005–1.199
	Constant	−7.973	3.158	6.374	0.012	0.000	
24 months	Total BDI-13	0.186	0.070	6.956	0.008	1.204	1.049–1.383
	Constant	−6.479	1.884	11.827	0.001	0.002	

Previous SA were coded 0 for “no” and 1 for “yes.” Degree of freedom = 1. ES, standard error; OR, odds ratio; CI, confidence interval.

### Potential predictors of SA at 12 months

No significant predictors were found at the 12 month mark.

### Potential predictors of SA at 18 months

At the 18 month follow-up, 10 out of 67 subjects (15.9%) have committed a new act of self-harm. Of the two potential predictors at 18 months, the presence of major depressive episodes did not show a significant relationship with SA. Only the total score on the TAS-20 questionnaire was retained (M = 63.9 vs. 71.1), *t*(62) = −2.24, *p* = 0.029, *d* = 0.82, with higher scores increasing the risk of attempting suicide (OR = 1.098, 95% CI = 1.01–1.20, *p* = 0.037) (Table 2). It explained 14% of the variance and correctly classified 82% of cases.

### Potential predictors of SA at 24 months

At the 24 month follow-up, 9 out of 82 subjects (10.9%) have committed a new act of self-harm. The BDI-13 questionnaire score (M = 19.1 vs. 27.2), the only potential predictor, did show a significant relationship with SA at 24 months, *t*(80) = −3.00, *p* = 0.004, *d* = 1.20. The model explained 23% of the variance and correctly classified 92% of the cases. The group at risk of PD with higher BDI-13 scores was more likely to SA (OR = 1.20, 95% CI = 1.05–1.38, *p* = 0.008) (Table 2).

## Discussion

This study is the first to prospectively explore predictors of SA among at-risk PD participants recruited from emergency departments. In the short term, a history of SA showed a 2- to 3-fold increase in the risk of recurrence. In the longer term, cognitive problems had a stronger influence, where each one-unit increment in TAS-20 and BDI-13 scores increased the risk of SA by 9.8 and 20.4%, respectively.

Previous SA predicted recurrence at 6 months. This is partly consistent with the study by Pompili and colleagues (31), who concluded that a previous SA remains a risk factor for recidivism for people with PD. We would have expected a stable effect, as this is one of the most robust factors in the general population (7, 32). However, SA, and other impulsive behaviors tend to decline over time among PD patients (33).

In the longer term, cognitive difficulties for subjects at risk of PD appear to be more important. Indeed, high mean scores found on the TAS-20 and BDI-13 were predictive of SA at 18 and 24 months, respectively. It was possible to reconfirm that alexithymia is a barrier to emotional regulation, which is paramount to moderating suicidal acts (14, 34). In light of this context and previous literature (35–40), we may inquire whether reducing alexithymia through enhancement of interpersonal interactions and adding expressive strategies could result in a decrease in the risk of suicide. Emotional dysregulation has previously been correlated many times with PDs (13), so much so that a vicious cycle seems to develop between emotional dysregulation, alexithymia, PD, and SI/SB. It might also be worth emphasizing the concept of “demoralization” (41, 42), which shares some similarities with depression and can be partially evaluated using the Beck Depression Inventory (BDI), and both could be relevant for clinical practices in emergency rooms and suicide risk prediction. Psychotherapeutic approaches aimed at improving reflective capacity would be advisable (43, 44). However, it should be noted that alexithymia explained only 14% of the variance in SA. The explained variance rose to 23% with the BDI-13, which is notable but still low. The latter percentage supports the notion that PD deserves more representation in suicide prevention efforts, since programs are often directed toward depression, although depression does not fully connect PD and SI/SB (45).

Our follow-up cohort of patients with personality disorders exhibits a significant likelihood of repeated instances of self-harm over a period of time, with rates of 22.9% at 6 months, 15.9% at 18 months, and 10.9% at 24 months. According to Baertschi and colleagues (46) the significance of personality in relation to suicidality is modest but noteworthy when viewed from the Interpersonal-Psychological Theory of Suicide perspective. As personality assessments are a common part of clinical practice, health care providers should take it into account as a supplementary tool to identify individuals who may have or show signs of suicidal thoughts.

The study by Grimholt and colleagues (17) showed that suicidal individuals with PD scored differently on the H and SIS scales compared to scales assessing other psychiatric disorders. In the present study, total H mean scores were also high, and SIS scores were moderate; when these scores were directly compared with each other, there was no relevance regarding the targeting of subjects at risk for PD who will SA. Regarding the SIS, it might be better to perform analyses by separating the objective section from the subjective section. Subjective willingness could be high, but the lack of intention could reflect a deficiency in interpersonal skills, preventing any appropriate social interaction normally expected during the staging of a SA.

In the general population, it has been well demonstrated that younger age, female sex, social isolation, and lower education are determining factors in those who SA (7–10). Our population at risk of PD did not replicate these findings. The subjects were mainly female (76%), but a significant interaction was not found. This may be a lack of sensitivity or a real absence of this sociodemographic effect.

Some limitations must be considered. The dissimilarities in the respondents across each follow-up could be the reason for the varying risk factors observed at each point. For example, a few respondents posted their responses at 6 and 18 months only, others posted their responses at 12 and 24 months, and so on. Furthermore, the small sample size may have influenced the quality of the multiple imputation outcomes. Most of the findings in this study were obtained through self-administered questionnaires. It is important to note that screening interviews may not be the most suitable method for making a diagnosis. We encourage readers to keep this in mind when evaluating our results. Also, the influence of psychiatric treatment on the risk of suicide cannot be ignored. However, due to the lack of follow-up data on the use of psychotropic drugs or psychotherapy, we were unable to assess their impact on outcomes. Last, the PDQ4-+ is a self-assessed PD screening tool that has average agreement with standard structured diagnostic interviews (e.g., SCID). It offers more false positives but rarely false negatives. Based on clinical reality in emergency centers, the PDQ4-+ remains an acceptable alternative when considering the limited time (47); it requires 10–15 min instead of 1 h–1.5 h. Overall, our results are exploratory and must be interpreted with caution. The study boasts several strengths, particularly its two-year follow-up and its status as one of the few studies to evaluate personality disorders in an emergency setting.

## Conclusion

In conclusion, these results question the validity of known predictors when prospectively applied to a sample of patients with self-poisoning SA. For individuals at risk of PD, these predictors seemed to have little influence. Screening PD in emergency departments is clearly needed and finding the best predictors of suicidal acts requires further exploration to guide the care offered. These data may advocate for the utilization of SI scales in emergency medical facilities.

## Data availability statement

The raw data supporting the conclusions of this article will be made available by the authors, without undue reservation.

## Ethics statement

The studies involving human participants were reviewed and approved by Toulouse’s University Hospital Center Institutional Ethical Committee (“Comité de Protection des Personnes Sud-Ouest et Outre-Mer II”). The patients/participants provided their written informed consent to participate in this study.

## Author contributions

LC was a major contributor in research implementation, interpreting data, and in writing the manuscript. MM performed statistical analyses and wrote the first version of this manuscript. AM co-directed the research. AY was a major contributor in writing the manuscript. PB was the main investigator of the research. All authors contributed to the article and approved the submitted version.

## Funding

This work was supported by Clinical Research Hospital Program of the French Ministry of Health (#2006-A00450-51).

## Conflict of interest

The authors declare that the research was conducted in the absence of any commercial or financial relationships that could be construed as a potential conflict of interest.

## Publisher’s note

All claims expressed in this article are solely those of the authors and do not necessarily represent those of their affiliated organizations, or those of the publisher, the editors and the reviewers. Any product that may be evaluated in this article, or claim that may be made by its manufacturer, is not guaranteed or endorsed by the publisher.
